# Decades of workplace health promotion research: marginal gains or a bright future ahead?

**DOI:** 10.5271/sjweh.3995

**Published:** 2021-10-31

**Authors:** Suzan Robroek, Pieter Coenen, Karen Oude Hengel

**Affiliations:** Department of Public Health Erasmus Medical Center Rotterdam Rotterdam, The Netherlands [email: s.robroek@erasmusmc.nl); Department of Public and Occupational Health Amsterdam Public Health Research Institute Amsterdam UMC Vrije Universiteit Amsterdam, Amsterdam, The Netherlands [email: p.coenen@amsterdamumc.nl]; Netherlands Organisation for Applied Scientific Research TNO Work Health and Technology Leiden, The Netherlands [email: karen.oudehengel@tno.nl] / Department of Public Health Erasmus Medical Center Rotterdam Rotterdam, The Netherlands [email: k.oudehengel@erasmusmc.nl]

## The potential of workplace health promotion

Unhealthy behaviors (eg, insufficient physical activity, an unhealthy diet, high alcohol intake and smoking) and obesity are risk factors for adverse health outcomes (1, 2), productivity loss due to presenteeism or sickness absence (3–6), and early exit from paid employment (7, 8). Poor health and unhealthy behaviors are more prevalent among workers from low socioeconomic positions (1, 2, 9), as are unemployment and work disability (10, 11). With a growing challenge in our societies to work longer, reflected in the increasing statutory retirement age in many European countries, an urgent need exists to enhance workers’ health to remain in paid employment. Given the profound socioeconomic inequalities in health behaviors, health, and participation in paid employment, this is particularly pressing among workers in lower socioeconomic positions.

The workplace is a promising setting for health promotion as workers spend a lot of time at work, and existing social networks for social support could be used to change behavior and enhance health. In the past decades, numerous workplace health promotion programs have been offered and evaluated regarding their (cost-)effectiveness. Workplace health promotion programs could be a way to improve workers’ health and can for example include elements of support, policies, or environmental changes to encourage healthy behavior. Traditionally, programs have focused on providing workers with advice on how to change their behavior. Such programs have been criticized because they do not take a broader perspective such as the environment (eg, workplace structures and conditions) into account (12). However, still many of these traditional programs are offered to employees and evaluated. The effects of such programs remain disappointing thus far. A recent review of reviews reported only small favorable long-term effects of workplace health promotion programs targeting physical activity and diet to reduce workers’ body weight (13). This is in line with findings from recent individual participant data meta-analyses of Dutch workplace health promotion programs that showed small and statistically non-significant decreases in unhealthy behaviors and body mass index (14, 15). In this editorial, we reflect on the body of research regarding workplace health promotion. Gaps in the literature will be described, most notably regarding (i) the need for more targeted workplace health promotion, (ii) a systems approach for workplace health promotion, and (iii) the delivery of workplace health promotion. We will conclude this editorial with future directions for workplace health promotion research.

## Gaps in the workplace health promotion literature

### Targeted workplace health promotion

A recent individual participant data meta-analysis showed that the effectiveness of workplace health promotion programs differed across target populations. Those programs focusing on indicated prevention (ie, on workers who are already at risk for unhealthy behavior, obesity or other health problems) were found to be more effective than universal prevention where a program is delivered to all workers within an organization (15). This is in contrast with the Geoffrey Rose paradigm, which implies that universal prevention, aimed at reducing the risk of an entire population, would be more effective from a public health perspective than interventions only targeting high-risk groups (16). However, as unhealthy behaviors and obesity are highly prevalent in the general population, the high-risk group consists of a large share of this population. It could therefore be argued that a targeted approach for workplace health promotion would be effective from a public health perspective as well.

In line with this and in an attempt to reduce socioeconomic health inequalities, effective interventions targeting workers in low socioeconomic position are needed. There are no indications that workplace health promotion programs differ in their effectiveness when delivered to different socioeconomic groups (14–16). However, a recent review on workplace health promotion showed that researchers substantially more often conduct studies on workplace health promotion among workers from higher compared to lower socioeconomic groups (17). This is striking because, as mentioned above, there is a particular need among workers in low socioeconomic groups to improve their health and reduce sickness absence and presenteeism. Researchers need to be encouraged to reach out to this group, even though it might be challenging.

### A systems approach to workplace health promotion

As universal behavioral prevention strategies on health behaviors or weight reduction in health promotion programs show little-to-no effect (13–15), it could be questioned whether and – if so – how workplace health promotion programs are justified. As unhealthy behaviors and obesity often coincide with pressing life struggles, including relational, emotional, financial and physical problems, single component interventions are unlikely to result in substantial changes (18). This notion is underlined by study results suggesting that improvements in health and productivity among workers is unlikely solely a behavioral issue. A recent article in this journal indicated that the work itself, rather than characteristics of the worker, account for one third of socioeconomic health differences (19). These recent findings reiterate discussion papers from decades ago arguing that health behavior change can hardly be reached by only providing people information and advice on how to become healthy (20). Approaches that combine individual interventions with changes in the environment and society are the most promising strategies to improve healthy behavior and reduce obesity (21). This means that more research is needed on the interplay between ‘causes of the causes’ of unhealthy behavior by trying to understand the ‘system’ in which people live and work. Only then, the structural determinants of health behavior among workers can be addressed. These so-termed system approaches are lacking within the occupation health setting or occur with only minimal changes in the environment.

### The delivery of workplace health promotion

The ineffectiveness of workplace health promotion programs cannot only be attributed to the target population or the content of the programs, but can also be the result of the lack of a clear implementation strategy and understanding of factors that may hinder or enable adequate uptake of workplace health promotion. In other words, what, why and how programs work in ‘real world’ settings.

Even though implementation research receives more and more attention in occupational health, it is still underrepresented in publications, including in this journal. Proper & van Oostrom (13) conclude that more research is needed on the factors that contribute to successful implementation of interventions. A systematic review showed room for improvement as initial participation levels in studies regarding workplace health promotion had a median participation level of only 33% (22). A meta-analysis indicated larger intervention effects among workers with higher program compliance (14), which emphasizes the importance of sustained participation with regard to the effectiveness. It is a particular challenge to reach workers with a lower socioeconomic position who typically work in blue-collar occupations and jobs involving difficult work circumstances such as shift work. A review on shift workers suggested that, to enhance participation, workplace health promotion programs should adopt more flexibility in the time and location of delivery of the program and time off (23). To reduce socioeconomic inequalities, in addition to delivering effective workplace health promotion programs, there is a need to gain more knowledge on implementation strategies to reach specifically workers with lower socioeconomic positions and to implement interventions in their context.

## Future directions of workplace health promotion research

Based on the knowledge gaps mentioned above, we propose the following research agenda concerning workplace health promotion. First, address underlying determinants of unhealthy behavior in workplace health promotion programs for workers with lower socioeconomic positions. Because of the persistent socioeconomic health inequalities and the low number of scientific studies conducted among workers with a lower socioeconomic position, there is undoubtedly a need for high quality studies on targeted interventions for these workers. These interventions should use approaches that go beyond a single behavioral component, for example a systems approach that considers underlying issues that coincide among workers with a low socioeconomic position (eg, unhealthy behaviors, unfavorable working conditions, health problems, and underlying social and financial issues).

Second, conduct process evaluations alongside effect evaluations to better understand how and why an intervention is (in-)effective. As aforementioned, although targeted interventions could be highly effective in the context of a research trial, it is important that they reach and retain the target group when implemented in a real-world setting. Designing the intervention and implementation strategies both deserve attention in the development phase of workplace health promotion programs to gain a better insight on what works for whom in which context and to make sure that successful workplace health promotion programs are sustainable in practice. To develop such implementation strategies, structured process evaluations to monitor the implementation alongside effect evaluations are needed (24). Although this suggestion is not new, and the number of process evaluations have increased in the past decades, publications of process evaluation still lag behind the publication of effects evaluations. A review showed that of 307 effect evaluations of workplace health promotion programs, only 27 (7.2%) published a process evaluation, which were moreover often of poor-to-average quality (25). We encourage researchers to conduct process evaluations and submit papers consisting of both a process and effect evaluation. In line with this, editors should also be more willing to publish such studies.

In conclusion, workplace health promotion programs thus far show marginal gains, as the effectiveness and implementation of traditional universal preventative workplace health promotion interventions are still disappointing. A drastic turnaround in occupational health research would be needed for us to have a bright future ahead with better tailoring and delivering interventions to the needs of the target group, in particular for workers with low socioeconomic positions.
